# Immunohistochemical evidence of tissue hypoxia and astrogliosis in the rostral ventrolateral medulla of spontaneously hypertensive rats

**DOI:** 10.1016/j.brainres.2016.09.012

**Published:** 2016-11-01

**Authors:** Tymoteusz Turlejski, Ibrahim Humoud, Roshni Desai, Kenneth J. Smith, Nephtali Marina

**Affiliations:** a Department of Neuroscience, Physiology and Pharmacology, University College London, UK; b Department of Neuroinflammation, Institute of Neurology, University College London, UK; c Clinical Pharmacology and Experimental Therapeutics, Division of Medicine, University College London, UK; d UCL Centre for Cardiovascular and Metabolic Neuroscience University College London, Gower Street, London WC1E 6BT, UK

**Keywords:** GFAP, glial fibrillary acidic protein, HIF-1α, hypoxia-induced factor 1α, MGV, Mean grey value, P_t_O_2_, tissue oxygen partial pressure, RVLM, rostral ventrolateral medulla, SHRs, spontaneously hypertensive rats, SNS, sympathetic nervous system, -ir, immunoreactive, Hypoxia, Arterial hypertension, Sympathetic nervous system, Astrogliosis

## Abstract

Increased activity of the sympathetic nervous system has been highlighted as a key factor that contributes to the development and maintenance of arterial hypertension. However, the factors that precipitate sustained increases in sympathetic activity remain poorly understood. Resting tissue oxygen partial pressure (P_t_O_2_) in the brainstem of anesthetized spontaneously hypertensive rats (SHRs) has been shown to be lower than in normotensive rats despite normal levels of arterial PO_2_. A hypoxic environment in the brainstem has been postulated to activate astroglial signalling mechanisms in the rostral ventrolateral medulla (RVLM) which in turn increase the excitability of presympathetic neuronal networks. In this study, we assessed the expression of indirect markers of tissue hypoxia and astroglial cell activation in the RVLM of SHRs and age-matched normotensive Wistar rats. Immunohistochemical labelling for hypoxia-induced factor-1α (HIF-1α) and bound pimonidazole adducts revealed the presence of tissue hypoxia in the RVLM of SHRs. Double immunostaining showed co-localization of bound pimonidazole labelling in putative presympathetic C1 neurons and in astroglial cells. Quantification of glial fibrillary acidic protein (GFAP) immunofluorescence showed relatively higher number of astrocytes and increased GFAP mean grey value density, whilst semi-quantitative analysis of skeletonized GFAP-immunoreactive processes revealed greater % area covered by astrocytic processes in the RVLM of adult SHRs. In conclusion, the morphological findings of tissue hypoxia and astrogliosis within brainstem presympathetic neuronal networks in the SHR support previous observations, showing that low brainstem P_t_O_2_ and increased astroglial signalling in the RVLM play an important role in pathological sympathoexcitation associated with the development of arterial hypertension.

## Highlights

• The rostral ventrolateral medulla of spontaneously hypertensive rats is hypoxic.
• Indirect markers of hypoxia were found in pre-sympathetic neurons and astrocytes.
• Astrogliosis is present in the rostral ventrolateral medulla of hypertensive rats.

## 1 Introduction

There is mounting evidence that increased sympathetic nervous system (SNS) activation is intricately linked to the development and maintenance of arterial hypertension, although the causative role is still controversial. Human studies have shown elevated plasma noradrenaline and increased vasoconstrictor sympathetic activity in individual organs such as the kidneys, heart and skeletal muscle in hypertensive patients (Esler et al., 1989, Esler et al., 2001, Esler et al., 1988) and it is estimated that over 50% of cases of essential hypertension are characterized by a substantial neurogenic component (Esler, 2010).

The mechanisms leading to increased SNS activity remain poorly understood, but recent studies have suggested that local abnormalities in oxygen homeostasis in the brainstem areas that generate sympathetic tone might play a substantial role. The brainstem vasculature in hypertensive patients and in the spontaneously hypertensive rat model (SHR) has been shown to be considerably narrow with a high resistance to blood flow and thus presympathetic networks that control blood pressure have been proposed to be hypoperfused (Cates et al., 2011, Cates et al., 2012a). There is also evidence that neurovascular coupling responses triggered by neuronal stimulation are significantly impaired in the brain of hypertensive subjects (Jennings et al., 2005, Calcinaghi et al., 2013, Iddings et al., 2001). Together, these abnormalities may cause important reductions in cerebral blood flow in hypertensive individuals. Direct oxygen measurements in the rostral ventrolateral medulla (RVLM) of anesthetized animals (via oxygen-sensitive fiber optic probe) have recently shown that local P_t_O_2_ levels in spontaneously hypertensive rats (SHRs) are significantly lower compared to control Wistar rats despite normal levels of arterial PO_2_ (Marina et al., 2015). This suggests that at resting conditions, the brainstem of hypertensive animals operates in hypoxic conditions.

Hypoxia is a well-known sympathoexcitatory stimulus leading to increased peripheral vascular resistance and increased arterial blood pressure (Sun and Reis, 1994). However, recent evidence indicates that sympathoexcitatory responses to hypoxia are indirectly mediated by prior activation of astroglial signalling mechanisms driven by the release of ATP and L-lactate (Marina et al., 2015). These (glio-) transmitters have been shown to increase neuronal excitability of pre-sympathetic C1 neurons of the RVLM, leading to increased sympathetic outflow and concomitant hypertension (Marina et al., 2015, Marina et al., 2016). However, the hypothesis that brainstem hypoxia and glial cell activation play a central role in sympathoexcitatory mechanisms associated with the development of hypertension still lacks important morphological clues. Thus, the purpose of this study was to compare the presence of tissue hypoxia and glial cell activation in the RVLM of SHRs and normotensive healthy controls.

## 2 Results

### 2.1 Detection of tissue hypoxia in the RVLM

**Fig. 1:**
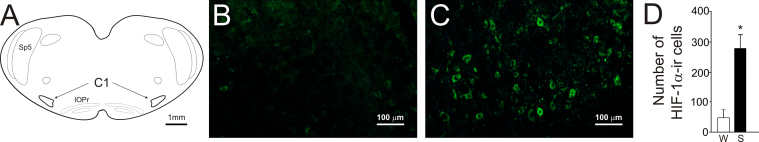
HIF-1α labelling in the brainstem of SHRs and Wistar rats. (A) Schematic representation of brainstem transverse section indicating the location of the C1 region. IOPr=Principal nucleus of the Inferior Olive; Sp5=Spinal trigeminal tract. Representative micrographs from a normotensive Wistar (B) and SHR (C). (D) Group data showing higher average number of cells expressing HIF-1α in the C1 region of adult SHRs compared to age-matched Wistar controls. Data are presented as mean±SEM. Unpaired *t*-test was used for statistical comparison. (*Significant difference compared with Wistar rats P=0.001).

**Fig. 2:**
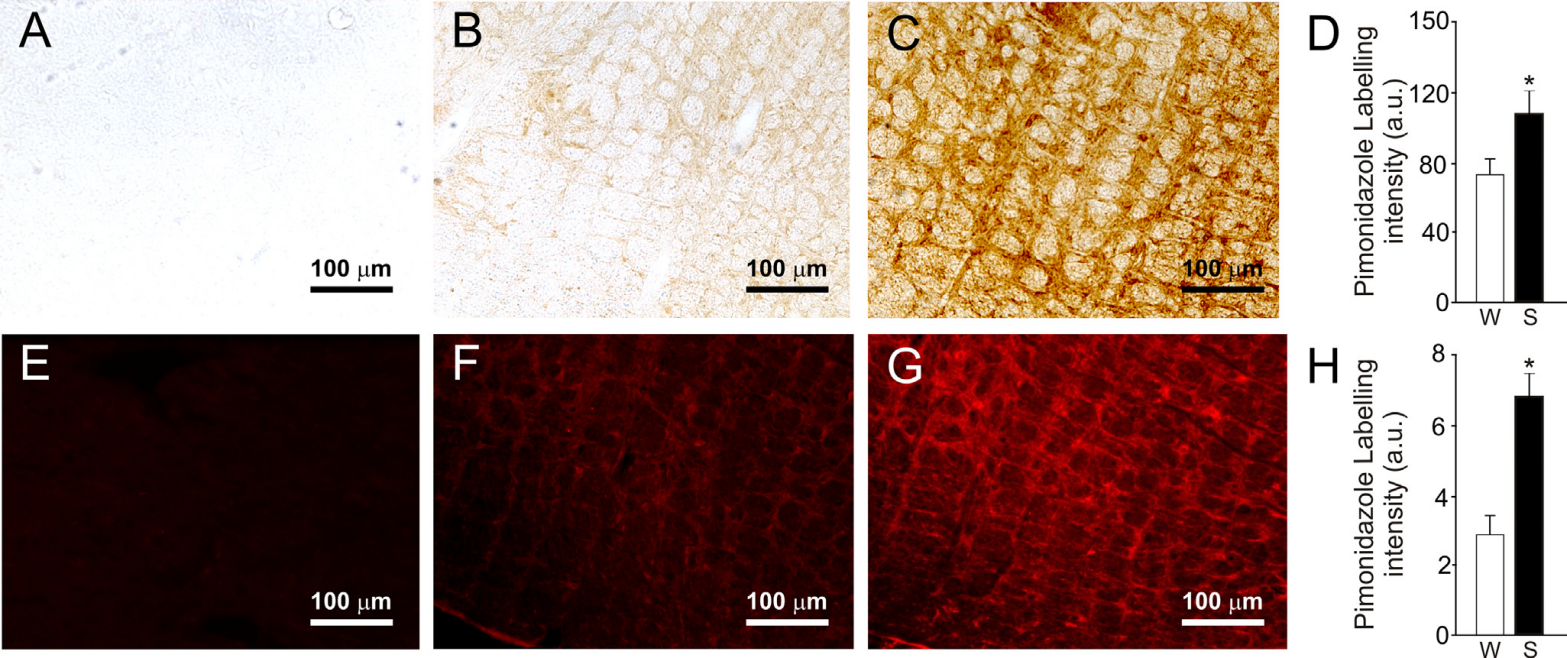
Pimonidazole labelling in the C1 area of SHR and Wistar rats. Representative micrographs of coronal brainstem sections labelled with immunoperoxidase (A–C) or immunofluorescence methods (E–G). Absence of labelling in negative controls is shown in (A) and (E). Micrographs corresponding to Wistar rats are shown in (B) and (F). Micrographs corresponding to SHRs are shown in (C) and (G). Group data showing increased pimonidazole immunoperoxidase labelling (D) and immunofluorescence labelling (H) in the C1 region of SHRs compared to age-matched Wistar controls. Data are presented as mean±SEM. Unpaired *t*-tests were used for statistical comparison. [*Significant difference compared with Wistar rats P=0.02 (D) and P=0.004 (H)].

**Fig. 3:**
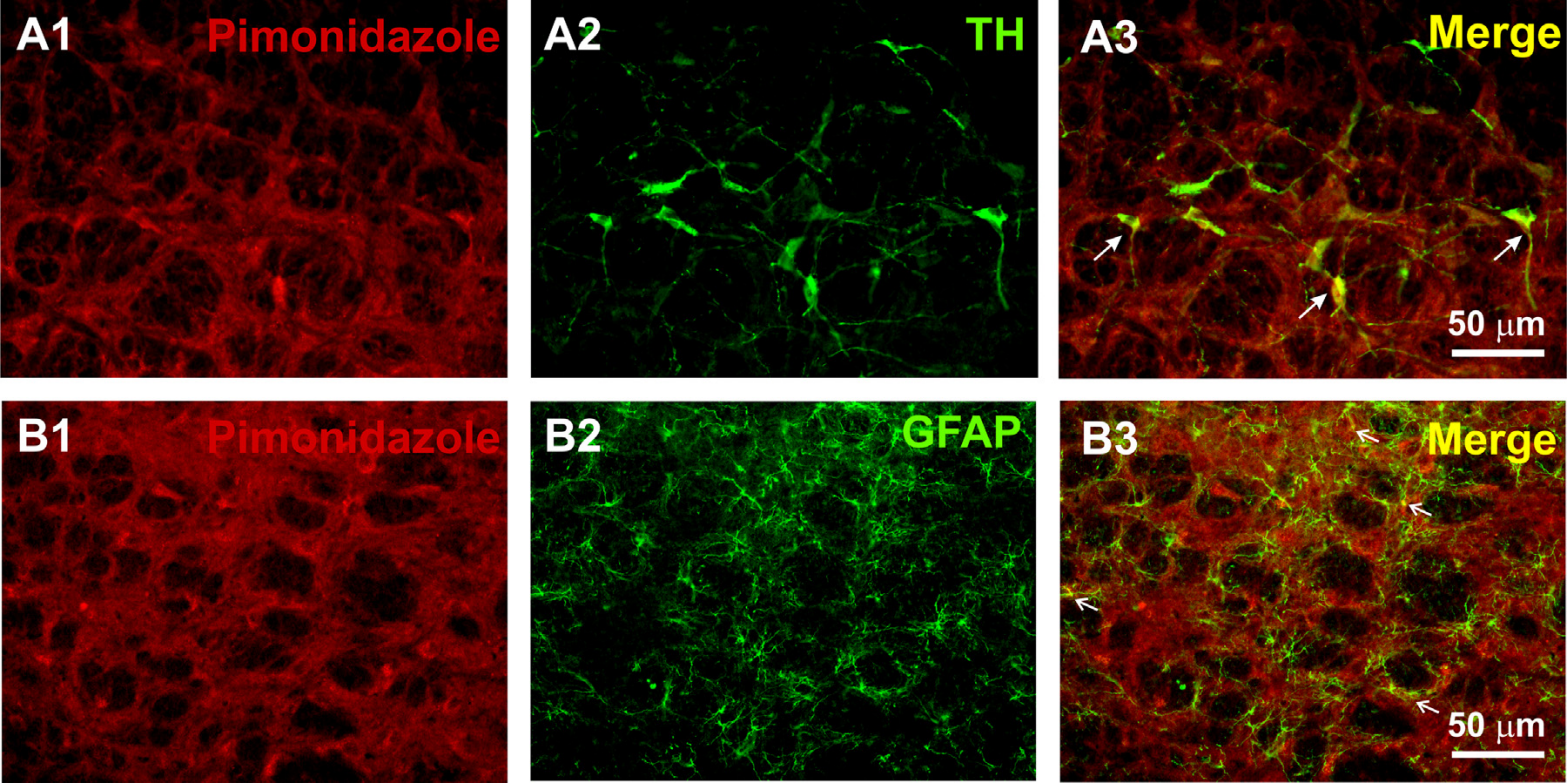
Pimonidazole labelling in specific cell types of the RVLM. (A1) and (B1), Representative micrographs of coronal brainstem sections showing pimonidazole immunofluorescent labelling in the C1 region of SHRs. Double immunostaining for tyrosine hydroxylase (TH; A2) and glial fibrillary acidic protein (GFAP; B2). Colour merge revealed co-localization of pimonidazole immunolabelling with the majority of TH-ir C1 neurons (A3, thick arrows) and with GFAP-ir astrocytes (B3, thin arrows).

We used two independent immunohistochemical methods to investigate the presence of tissue hypoxia in the brainstem of hypertensive rats. First, abundant hypoxic cells were identified in the C1 region of naïve SHRs and to a much lesser extent in Wistar rats immunolabelled for HIF-1α. The labelling was mainly located within the cytoplasm of unidentified cellular structures within the RVLM. We found greater numbers of HIF-1α-immunoreactive (-ir) cells in the C1 region of SHRs compared to Wistar rats (276.4±49.8 cells in SHRs vs 53.1±23.3 cells in Wistar rats, p<0.001, n=5; Fig. 1). Second, in animals that received a systemic administration of pimonidazole, we detected clear labelling for bound pimonidazole in the brainstem of hypertensive rats and also, to a much lesser extent in control Wistar rats. The presence of bound pimonidazole adducts revealed by either immunoperoxidase or immunofluorescence methods was characterized by diffuse labelling in the RVLM parenchyma. Optical densitometric analysis of bound pimonidazole revealed increased immunoperoxidase labelling in the RVLM of SHRs compared to Wistar rats (113.5±11.3 a.u. in SHRs vs 76±15.6 a.u. in Wistar rats, p=0.02; Fig. 2A–D). Similar differences were observed in tissue labelled with immunofluorescence methods (6.5±0.6 a.u. in SHRs vs 3.1±0.5 a.u. in Wistar rats, p=0.004; Fig. 2E–H). Double immunofluorescence labelling for pimonidazole and cell-specific markers for C1 catecholaminergic neurons and astroglial cells in the brainstem of SHRs revealed co-localization in the majority of tyrosine hydroxylase- (TH-) positive neurons and GFAP-positive astroglial cells residing within the C1 area (Fig. 3).

### 2.2 Glial cell activation in the RVLM of SHRs

**Fig. 4:**
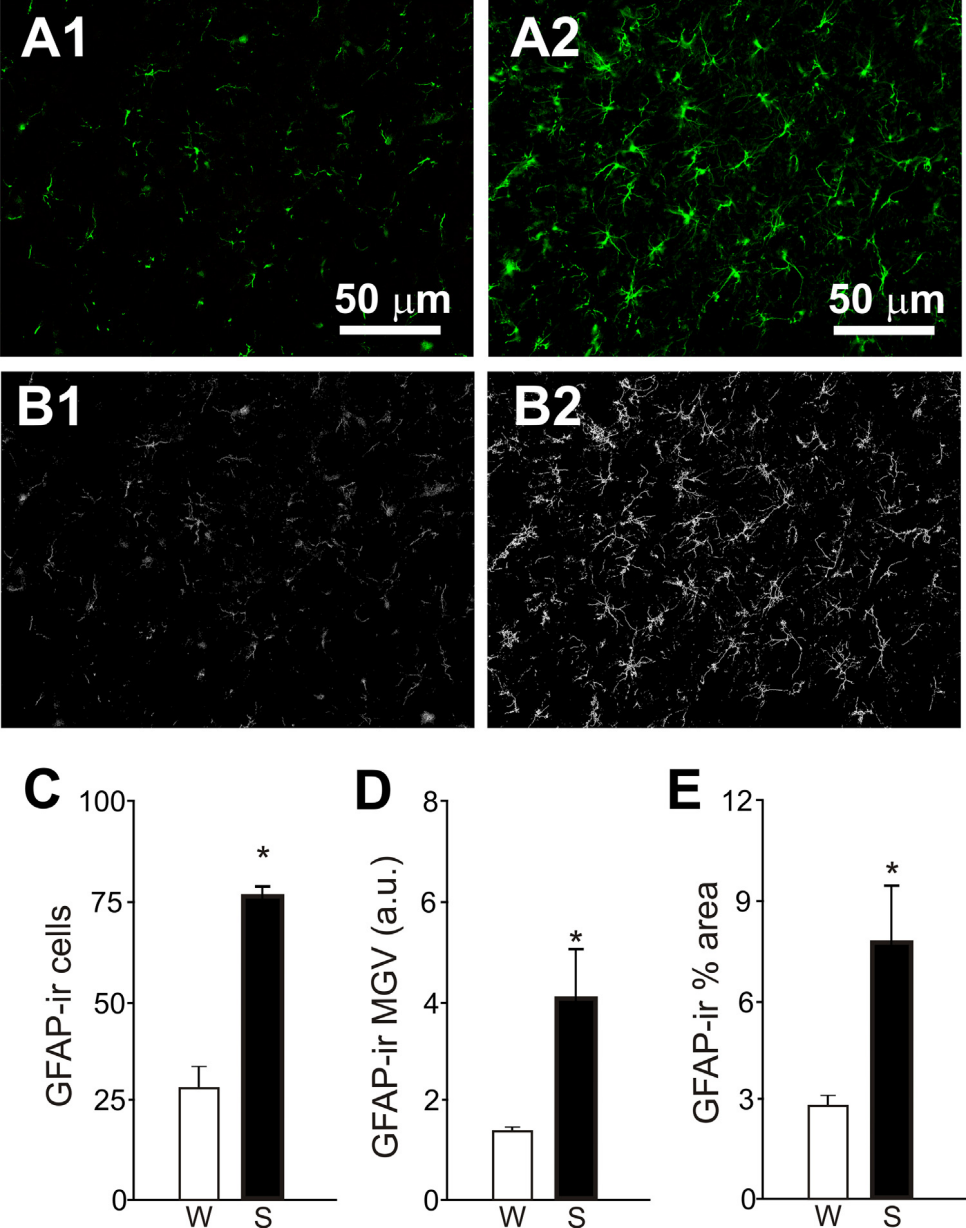
Immunolabelling of astrocytes in the RVLM rats. Representative micrographs of coronal brainstem sections labelled immunohistochemically to detect glial fibrillary acidic protein (GFAP) in the C1 region of SHRs (A1) and Wistar rats (A2). Skeletonized images used for the analysis are shown in B1 and B2, respectively. Group data showing increased number of GFAP-ir astrocytes (C), increased mean grey value (MGV) (D) and larger % area covered by GFAP-ir astroglial processes (E) in the in the C1 region of SHRs in comparison to Wistar rats. Data presented as mean±SEM. Unpaired *t*-test was used for statistical comparisons. [*Significant difference compared with Wistar rats P=0.004 (C), P=0.02 (D) and P=0.01 (E)].

As increased astroglial signalling in the brainstem has been shown to play a significant role in sympathoexcitatory mechanisms associated with the development of arterial hypertension, we sought to detect anatomical evidence of glial cell activation in the RVLM of SHR (Fig. 4). Quantification of GFAP-immunofluorescence in the C1 region revealed increased number of GFAP-ir astrocytes in SHRs compared to Wistar rats (76.5±2.7 cells in SHRs vs 27.9±8.1 cells in Wistar rats, p=0.004; Fig. 4C) as well as increased mean grey value of GFAP staining (MGV, 4.1±0.9 a.u. in SHRs vs 1.4±0.1 a.u. in Wistar rats, p=0.02; Fig. 4D). Semi-quantitative analysis of GFAP-ir skeletonized processes showed greater % area occupied by astrocytic processes in the C1 region of SHRs (7.5±2.1% in SHRs vs 2.8±0.3% in Wistar rats, p=0.001; Fig. 4E).

## 3 Discussion

In this study we sought to obtain immunohistochemical evidence that in the SHR, brainstem presympathetic networks that control arterial blood pressure are embedded in a hypoxic microenvironment. First, using two independent immunolabelling methods, we confirmed the presence of tissue hypoxia in the RVLM of the SHR. Next, we found which cells in the RVLM show this hypoxic phenotype. Finally, we determined the presence of astrogliosis in the RVLM of SHRs using three independent methods for semi-quantitative assessment of GFAP immunolabelling.

### 3.1 Tissue hypoxia

The brain is highly vulnerable to the harmful effects of sustained arterial hypertension because it is dependent on luxuriant blood flow for its energy requirements. Hypertension accelerates atheroschlerotic plaque formation in the cerebrovascular tree which may lead to microvascular brain damage, stenosis and stroke. In the brainstem, hypertension has been shown to induce considerable remodelling of the basilar artery and increased vascular resistance and together, these factors are believed to compromise brainstem perfusion (Cates et al., 2012a, Cates et al., 2012b, Cates et al., 2011, Dickinson and Thomson, 1960). In a recent study, we showed that P_t_O_2_ measurements in the C1 region of isoflurane-anesthetized SHRs are significantly lower compared to normotensive Wistar rats, despite normal levels of arterial PO_2_ (Marina et al., 2015). Paradoxically, RVLM hypoxia was exacerbated when arterial blood pressure was acutely reduced to levels comparable to those seen in normotensive animals (Marina et al., 2015). Central hypoxia is a powerful sympathoexcitatory stimulus (Sun and Reis, 1994) and in subjects with arterial hypertension, this mechanism is believed to induce compensatory increases in sympathetic outflow and systemic blood pressure in order to overcome excessive resistance at the level of the basilar artery with the aim of preventing the exacerbation of brainstem hypoxia (Cushing mechanism) (Cates et al., 2012a, Cates et al., 2012b, Cates et al., 2011, Marina et al., 2015).

In our study, immunolabelling of tissue hypoxia markers in the C1 region was consistently higher in SHRs. Our results show that HIF-1α immunostaining was localized in cells that judged by their size and morphology, resemble neuronal structures. However, due to technical reasons we were unable to demonstrate the identity of these cells. On the other hand, bound pimonidazole immunofluorescence in the RVLM of SHRs was found in cell types identified as catecholaminergic neurons and in astroglial cells suggesting that both cell groups are indeed hypoxic. In the RVLM, pre-sympathetic neurons were believed to be directly sensitive to hypoxia (Dampney and Moon, 1980, Sun and Reis, 1994). However, recent evidence has shown that presympathetic neurons do not respond directly to hypoxia, but sense ATP and lactate released by neighbouring astrocytes (Marina et al., 2015). Brainstem astrocytes are physiological oxygen sensors in the brain, responding to decreases in PO_2_ a few mmHg below normal brain oxygenation with elevations in intracellular [Ca^2+^], leading to ATP and lactate release which in turn activate presympathetic neurons resulting in increased renal sympathetic activity and arterial blood pressure (Marina et al., 2015, Angelova et al., 2015, Karagiannis et al., 2016). We have shown that accumulation of ATP in the RVLM contributes to sustained sympathoexcitation leading to the development of systemic hypertension (Marina et al., 2015). To interfere with ATP-mediated signalling, we used a lentiviral vector to express a potent ectonucleotidase—transmembrane prostatic acid phosphatase (TMPAP). TMPAP promotes breakdown of extracellular ATP and expression of TMPAP within RVLM pre-sympathetic circuits reduces sympathoexcitation and the development of arterial hypertension in the SHRs (Marina et al., 2013, Marina et al., 2015). Thus, we believe that a local hypoxic environment in the RVLM triggers astroglial cell activation leading to SNS activation and compensatory hypertension (Cushing mechanism), the purpose of which is to preserve brain perfusion. However, this mechanism has the potential to become maladaptive resulting in systemic hypertension (the so-called “selfish brain” hypothesis) (Rodbard and Stone, 1955, Dickinson and Thomson, 1960, Paton et al., 2009, Cates et al., 2011, Marina et al., 2015).

### 3.2 Astrogliosis in the RVLM

Our results show the presence of glial cell activation in the C1 region of SHRs, characterized by increased number of GFAP-ir astrocytes, increased GFAP mean grey value intensity and increased % C1 area occupied by GFAP-ir astrocytic processes. Astrocytes residing in the RVLM have been shown to have a profound influence on the activity of bulbospinal sympathoexcitatory neurons. Optogenetic activation of RVLM astrocytes in vitro (transduced to express channelrhodopsin2) increases the excitability of catecholaminergic presympathetic neurons which belong to the C1 group (Marina et al., 2013). These responses were found to be significantly attenuated in the presence of ATP-degrading enzyme apyrase, indicating a pivotal role for ATP in astrocyte-mediated activation of presympathetic neurons (Marina et al., 2013). Furthermore, optogenetic activation of ChR2-expressing astrocytes within the RVLM in anesthetized rats produces substantial increases in renal sympathetic nerve activity, heart rate and arterial blood pressure (Marina et al., 2013). Increased expression of GFAP is indicative of reactive astrogliosis – as defined by glial cell activation due to injury or pathological processes including hypoxia, infection and trauma (Eng and Ghirnikar, 1994). Thus, our immunohistochemical findings provide morphological evidence of astrogliosis in the RVLM of SHRs which is consistent with our previous observations showing increased astroglial signalling in the RVLM of SHRs (Marina et al., 2015). However, the mechanisms leading to astrogliosis in the brainstem of hypertensive rats as well as the biological effects of reactive astrocytes surrounding presympathetic networks that control arterial blood pressure are unknown. It remains to be determined whether gliotransmitter release mechanisms are exaggerated in reactive astrocytes.

In summary, the data obtained in the present study supports our hypothesis that tissue hypoxia in the RVLM is a key element of the Cushing mechanism and the morphological finding of astrogliosis in the C1 region of the SHR provides an anatomical substrate that links cerebrovascular abnormalities and brainstem hypoperfusion with increased SNS activity. Thus, we propose that sustained activation of RVLM astrocytes (either in response to localized hypoxia, inflammation, reactive oxygen species, etc.) facilitates the stimulation of presympathetic C1 neurons, leading to maladaptive increases in SNS activity and elevated arterial blood pressure (Marina et al., 2016). Future studies should evaluate whether sex has an impact on these pathophysiological mechanisms.

## 4 Experimental procedure

All animal experimentations were performed in accordance with the European Commission Directive 86/609/EEC (European Convention for the Protection of Vertebrate Animals used for Experimental and Other Scientific Purposes) and the United Kingdom Home Office (Scientific Procedures) Act (1986) and ARRIVE (Animal Research: Reporting of *In Vivo* Experiments) guidelines with project approval from the UCL Institutional Animal Care and Use Committee.

### 4.1 Histological assessment of tissue hypoxia and glial cell activation

Brainstem tissue from naïve adult (15 weeks) male SHRs (n=5) and age- and sex-matched normotensive Wistar rats (n=5) was used for immunohistochemical detection of HIF1α (indirect marker of tissue hypoxia) and GFAP (astroglial marker). In a separate group of rats, tissue hypoxia was assessed by immunodetection of bound pimonidazole HCl protein-thiol adducts (Davies et al., 2013). Briefly, adult (15 weeks) male SHRs (n=5) and age- and sex-matched Wistar rats (n=4) were injected with pimonidazole (60 mg/kg i.p. dissolved in sterile saline, Hypoxyprobe, HPI Inc., USA) 4 h before terminal anaesthesia for intracardiac fixation. In conditions of hypoxia, pimonidazole is enzymatically reduced and it binds to tissue where it can be visualized using immunohistochemistry and it has been previously shown to be a reliable marker of hypoxia in the central nervous system (Davies et al., 2013, Arteel et al., 1998).

Animals were deeply anesthetized with urethane (1.9 g kg^−1^ i.p) and transcardially perfused with ice-cold 0.9% sodium chloride solution followed by 4% paraformaldehyde at 4 °C. Brains were extracted, postfixed in PFA overnight, and cryoprotected in 30% sucrose for 48 h at 4 °C. Brainstems were sectioned with a cryostat (Bright instruments, UK) along the rostro-caudal extent of the RVLM (starting caudally from the area postrema (AP) and finishing approximately 2 mm rostral to the AP). Transverse sections (30 µm) were collected serially in 6 well plates containing anti-freeze solution (Marina et al., 2002) and stored at −21 °C until further processing.

### 4.2 Immunohistochemistry

HIF1-α, pimonidazole, GFAP, and TH were detected by immunofluorescence methods. Negative controls were performed for each antibody by omitting the primary antibody. Free-floating sections were washed 3 times for 5 min in 0.1 M phosphate-buffered saline (PBS) and where necessary, were incubated in special buffers for antigen retrieval [DAKO® Target retrieval solution (pH 6.0) for HIF1-α and sodium borohydride (1 mg/ml in PBS) for Pimonidazole]. Brainstem sections were then incubated in blocking buffer for 1 h, to minimise non-specific binding of the antibody and for cell membrane permeabilisation (10% goat serum in 0.1% Triton in PBS for HIF1-α and 0.25% casein in 0.1% Triton in PBS for Pimonidazole, TH and GFAP). Tissue was incubated in primary antibodies diluted in blocking buffer for 48 h at 4 °C [Rabbit anti-HIF1-α (1:500, Novus-Bio); Mouse anti-Pimonidazole (1:200, HPI Inc); Rabbit anti-Tyrosine Hydroxylase (1:500, Abcam); Rabbit anti-glial fibrillary acidic protein GFAP (1:500, Dako)]. Tissue was washed 3 times with PBS and subsequently incubated in secondary antibodies for 2 h [Alexa Fluor 488 Donkey anti-rabbit or Alexa Fluor 568 Donkey anti-mouse (1:1000, Molecular probes)]. For double immunostaining protocols, two separate sets of serial histological sections were first stained for pimonidazole, followed by either TH or GFAP. After 3 final washes in PBS, the tissue was mounted on glass slides and cover-slipped with Fluoroshield® (Sigma).

Pimonidazole binding was also detected in a separate set of serial sections by the immunoperoxidase method. Endogenous peroxidase activity was eliminated with 0.3% H_2_O_2_ diluted in methanol. The sections were washed in PBS and incubated in primary antibody followed by biotinylated horse anti-mouse secondary antibody (1:200, Vector Laboratories). The Avidin-Biotin complex (ABC) protocol was followed using Vectastain® Elite ABC kit (Vector Laboratories) with 3-3′ Diaminobenzidine as the chromogen. The tissue was mounted on glass slides and air-dried overnight, followed by dehydration in ethanol (70%, 90%, 100%) and xylene (twice each and for 2 min per step) and cover-slipped with DPX (Thermo Scientific™).

### 4.3 Image analysis

Digital images were captured using a Leica DM2000 fluorescence microscope (Leica Microsystems, Germany) coupled to a Retiga 3000C camera (Q Imaging, Canada). The C1 region was defined as the area located near the ventral medullary surface between the principal nucleus of the inferior olive (IO Pr) and the spinal trigeminal tract (Sp5). Two images were captured per slice with either 10× or 20× objectives (one from each ipsilateral C1 region) with a total of 5 sections covering the rostro-caudal extent of the C1 area (approximately between 11.60 mm and 12.80 mm caudal to Bregma). All images were captured by a blind investigator during the same session and using the same camera settings. Images were analysed using WCIF ImageJ software as follows:

### 4.4 HIF1-α cell counts

Micrographs were captured at 10X and converted to 16-bit scale. The cell counter plugin was used to quantify the total number of labelled cells in the C1 region regardless of their size or label intensity.

### 4.5 Pimonidazole immunoperoxidase intensity

Micrographs were captured using a 10× objective. Images with white DAB signal on a black background were obtained after the original image was inverted and converted into 8-bit scale. Mean grey value (MGV) was determined using the analysis function in imageJ and the value was corrected by subtracting the level of background staining from an image of a slice incubated in the absence of primary antibody. Data are expressed as average pimonidazole labelling in arbitrary units (a.u.).

### 4.6 Pimonidazole immunofluorescence intensity

Images were captured at 10× magnification and converted to 8-bit scale. MGV was calculated as a measure of immunofluorescence intensity of pimonidazole. Data are shown as average intensity of immunofluorescence expressed in a.u.

### 4.7 GFAP analysis

Micrographs were captured at 20× and converted into 16 bit scale. The cell counter plugin was used to quantify manually the total number of labelled cells per image. Only cell bodies clearly identified by GFAP-immunofluorescence regardless of their label intensity, size and number and length of astroglial processes were included in the analysis. Grey scale images were then transformed into binary images using the threshold function and were subsequently skeletonized using *Skeletonize 2D/3D* plugin. MGV and % area occupied by GFAP-ir skeletonized processes were quantified using the analysis function.

### 4.8 Data analysis

Immunohistochemical levels of expression of tissue hypoxia and glial cell activation markers are presented as mean±SEM and were compared using unpaired, two-sample, two-tailed Student's *t*-test. The difference was deemed statistically significant at the level of p<0.05.

## Funding sources

This work was supported by a British Heart Foundation Intermediate Basic Research Science Fellowship for Nephtali Marina (Grant number FS/13/5/29927); the Multiple Sclerosis Society UK (979/12); National Multiple Sclerosis Society USA (RG 5056A4); and Rosetrees Trust, United Kingdom (A997).
